# Supplementary material for: Impact of Acid Suppression Therapy on Renal and Survival Outcomes in Patients with Chronic Kidney Disease: A Taiwanese Nationwide Cohort Study
Source: J Clin Med. 2022 Sep 23;11(19):5612. doi: 10.3390/jcm11195612 (PMC9570958; doi:10.3390/jcm11195612)
Supplement: Supplementary file 1 [file jcm-11-05612-s001.zip › jcm-1872732-supplementary.pdf]

Table S1. Association between individual acid suppressant and study outcomes

Taking controls as the reference	ESRD events, %	Adjusted HR* (95% CI)	*p*-value	Overall mortality event, %	Adjusted HR# (95% CI)	*p*-value
Individual PPI						
Omeprazole	105 (9.5)	1.28 (0.89-1.83)	0.19	583(52.8)	1.99 (1.70-2.33)	<0.0001
Pantoprazole	81 (6.9)	1.68 (1.04-2.72)	0.034	427 (36.4)	2.07 (1.70-2.52)	<0.0001
Lansoprazole	68 (5.9)	0.94 (0.59-1.50)	0.80	269 (23.3)	1.57 (1.26-1.96)	<0.0001
Rabeprazole	15 (3.1)	1.69 (0.53-5.46)	0.38	58 (12.1)	1.06 (0.66-1.70)	0.82
Esomeprazole	91 (5.4)	0.99 (0.68-1.44)	0.96	480 (28.6)	1.75 (1.46-2.09)	<0.0001
Individual H2RA						
Cimetidine	59 (1.7)	0.34 (0.24-0.48)	<0.0001	469 (13.6)	0.62 (0.54-0.71)	<0.0001
Ranitidine	12 (2.1)	0.36 (0.11-1.18)	0.09	113 (20.0)	0.83 (0.61-1.12)	0.22
Famotidine	25 (1.6)	0.29 (0.17-0.48)	<0.0001	206 (13.1)	0.57 (0.46-0.70)	<0.0001

Abbreviations: the same as Tables 1-3.

*Adjusted for all covariates (age per year, sex, comorbidities, number of medical visits, NSAID, and ACEI/ARB) and competing mortality.

#Adjusted for all covariates (age per year, sex, comorbidities, number of medical visits, NSAID, and ACEI/ARB).

Table S2. Multivariable stratified analyses for the association between acid-suppressant use and study outcomes. Each factor was adjusted for all other factors listed in Table 1. The adjusted hazard ratio (aHR) of end-stage renal disease (ESRD) was estimated in the presence of competing risk

	ESRD				Overall mortality			
	PPI (*vs.* Control)		H2RA (*vs.* Control)		PPI (*vs.* Control)		H2RA (*vs.* Control)	
Variable	aHR	95% CI	aHR	95% CI	aHR	95% CI	aHR	95% CI
Age (year)								
18-55	1.00	0.68-1.48	0.35	0.22-0.56	2.22	1.72-2.86	0.38	0.27-0.53
≥56	1.26	0.94-1.69	0.43	0.30-0.61	1.73	1.54-1.94	0.69	0.61-0.78
Sex								
Male	0.96	0.71-1.29	0.43	0.31-0.60	1.77	1.56-2.01	0.64	0.55-0.74
Female	1.54	1.04-2.26	0.32	0.19-0.54	1.97	1.64-2.36	0.62	0.51-0.76
Coronary heart disease								
No	1.12	0.88-1.44	0.39	0.29-0.52	1.91	1.71-2.14	0.65	0.57-0.73
Yes	1.62	0.66-3.99	0.63	0.22-1.78	1.25	0.94-1.67	0.57	0.42-0.77
Diabetes								
No	1.12	0.83-1.52	0.32	0.22-0.48	1.84	1.64-2.07	0.64	0.56-0.73
Yes	1.26	0.86-1.85	0.53	0.35-0.80	1.71	1.37-2.14	0.63	0.50-0.80
Hypertension								
No	0.95	0.67-1.35	0.33	0.22-0.50	2.05	1.77-2.38	0.66	0.56-0.78
Yes	1.49	1.08-2.06	0.52	0.35-0.75	1.56	1.34-1.81	0.60	0.51-0.71
Acid peptic disease								

No	1.12	0.89-1.42	0.40	0.30-0.52	1.82	1.64-2.02	0.63	0.56-0.71
Yes	9.78	1.01-94.51	0.71	0.05-10.51	2.20	1.03-4.71	1.13	0.53-2.43
Chronic liver disease								
No	1.15	0.91-1.46	0.41	0.31-0.55	1.80	1.62-2.00	0.64	0.57-0.72
Yes	0.99	0.19-5.11	0.10	0.01-1.52	2.46	1.45-4.16	0.54	0.29-1.02
ACEI/ARB								
No	1.18	0.90-1.55	0.32	0.22-0.47	1.85	1.66-2.08	0.57	0.50-0.65
Yes	1.40	0.79-2.47	0.58	0.31-1.07	1.76	1.29-2.41	0.76	0.55-1.06
NSAID								
No	1.18	0.92-1.51	0.39	0.28-0.53	1.56	1.39-1.76	0.65	0.57-0.74
Yes	0.67	0.27-1.61	0.24	0.09-0.60	2.48	1.80-3.42	0.75	0.54-1.05
No. of medical visits								
1-11	0.94	0.66-1.35	0.36	0.23-0.56	1.93	1.61-2.32	0.64	0.52-0.79
≥12	1.35	0.98-1.84	0.44	0.30-0.64	1.78	1.57-2.02	0.64	0.56-0.74

Abbreviations: the same as Tables 1-4.

Table S3. Additional sensitivity analyses in our CKD population

	ESRD*			Overall mortality		
	aHR	95% CI	*P*-value	aHR	95% CI	*P*-value
**Model 1.** Adding anti-platelet and anti-lipid drugs into the original regression model listed in Table 2						
Control	1.00	Reference		1.00	Reference	
PPI cohort	1.14	0.90-1.45	0.26	1.83	1.65-2.02	<0.0001
H2RA cohort	0.40	0.30-0.53	<0.0001	0.64	0.57-0.72	<0.0001
**Model 2.** Excluding the same CKD year from matching variables, redefining comorbidity diabetes and hypertension by ICD-9/10-CM codes or anti-diabetic and antihypertensive drug codes, and adding anti-platelet and anti-lipid drugs into the original regression model listed in Table 2						
Control	1.00	Reference		1.00	Reference	
PPI cohort	0.99	0.83-1.20	0.96	1.46	1.35-1.58	<0.0001
H2RA cohort	0.41	0.33-0.50	<0.0001	0.50	0.46-0.55	<0.0001
**Model 3.** Only comparing two cohorts (PPI *vs* H2RA) in the original propensity-matched CKD cohort						
PPI cohort	1.00	Reference		1.00	Reference	
H2RA cohort	0.35	0.27-0.45	<0.0001	0.36	0.33-0.40	<0.0001
**Model 4.** Adding two comorbidities glomerulonephritis and acute tubular necrosis and included them into propensity score matching and the original regression model listed in Table 2						
Control	1.00	Reference		1.00	Reference	
PPI cohort	1.16	0.92-1.47	0.21	1.83	1.65-2.03	<0.0001
H2RA cohort	0.40	0.30-0.52	<0.0001	0.64	0.57-0.72	<0.0001

Abbreviations: the same as Tables 1-4.

*in the presence of competing mortality.

Table S4. Association between cumulative define daily dose (cDDD) by 15, 30, and 45 of PPI and study outcomes

	ESRD			Overall mortality			CV mortality			Non-CV mortality		
Taking controls as the reference	events (%)	aHR* (95% CI)	*p*-value	events (%)	aHR# (95% CI)	*p*-value	events (%)	aHR# (95% CI)	*p*-value	events (%)	aHR# (95% CI)	*p*-value
cDDD≤15 (n=1 674)	131 (7.8%)	1.36 (0.95-1.94)	0.1	753 (45.0%)	2.33 (2.00-2.72)	<0.0001	102 (6.1%)	1.92 (1.21-3.06)	0.006	651 (38.9%)	2.38 (2.01-2.80)	<0.0001
15<cDDD≤ 30 (n=550)	27 (4.9%)	0.72 (0.35-1.46)	0.36	141 (25.6%)	1.74 (1.25-2.41)	0.001	15 (2.7%)	2.42 (0.90-6.49)	0.08	126 (22.9%)	1.66 (1.17-2.35)	0.005
30<cDDD≤ 45 (n=435)	17 (3.9%)	0.91 (0.39-2.10)	0.82	90 (20.7%)	1.94 (1.29-2.92)	0.002	9 (2.1%)	1.57 (0.46-5.35)	0.47	81 (18.6%)	2.02 (1.31-3.13)	0.002
45<cDDD (n=1 702)	95 (5.6%)	1.33 (0.90-1.96)	0.15	331 (19.4%)	1.26 (1.05-1.52)	0.015	31 (1.8%)	1.20 (0.67-2.17)	0.54	300 (17.6%)	1.27 (1.04-1.55)	0.019

Abbreviations: CV, cardiovascular; the same as Tables 1-4.

*Adjusted for all covariates (age per year, sex, comorbidities, number of medical visits, NSAID, and ACEI/ARB) and competing mortality.

#Adjusted for all covariates (age per year, sex, comorbidities, number of medical visits, NSAID, and ACEI/ARB).

Table S5. Hazard ratios (HRs) for end-stage renal disease (ESRD) and overall mortality in three cohorts roughly by CKD stages 1-4 *vs.* stage 5

	Crude			Adjusted		
Outcome	HR	95% CI	*p*-value	HR	95% CI	*p*-value
**Before propensity score matching**						
Stages 1-4 CKD population						
ESRD* (event number/total n)						
Control (4 542/46 949)	1.00	Reference		1.00	Reference	
PPI cohort (2 429/7 106)	2.47	2.24-2.73	<0.0001	0.85	0.74-0.97	0.014
H2RA cohort (4 495/48 586)	0.57	0.53-0.62	<0.0001	0.43	0.39-0.47	<0.0001
Overall mortality# (event number/total n)						
Control (5 432/46 949)	1.00	Reference		1.00	Reference	
PPI cohort (2 769/7 106)	5.91	5.63-6.20	<0.0001	2.23	2.12-2.35	<0.0001
H2RA cohort (5 035/48 586)	0.95	0.91-0.99	0.01	0.75	0.72-0.78	<0.0001
Stage 5 CKD population						
ESRD* (event number/total n)						
Control (106/123)	1.00	Reference		1.00	Reference	
PPI cohort (10/15)	0.82	0.35-1.89	0.64	0.69	0.25-1.92	0.47
H2RA cohort (15/23)	0.59	0.33-1.07	0.08	0.49	0.27-0.86	0.014
Overall mortality# (event number/total n)						
Control (46/123)	1.00	Reference		1.00	Reference	
PPI cohort (7/15)	1.91	0.70-5.20	0.21	4.20	1.44-12.20	0.008
H2RA cohort (7/23)	0.85	0.37-1.95	0.71	1.88	0.73-4.87	0.19
**After propensity score matching**						

Stages 1-4 CKD population						
ESRD* (event number/total n)						
Control (136/4 358)	1.00	Reference		1.00	Reference	
PPI cohort (263/4 349)	2.01	1.64-2.47	<0.0001	1.15	0.90-1.45	0.26
H2RA cohort (81/4 361)	0.58	0.44-0.77	<0.0001	0.41	0.31-0.54	<0.0001
Overall mortality# (event number/total n)						
Control (136/4 358)	1.00	Reference		1.00	Reference	
PPI cohort (263/4 349)	2.54	2.31-2.79	<0.0001	1.83	1.65-2.03	<0.0001
H2RA cohort (81/4 361)	0.98	0.88-1.09	0.71	0.64	0.57-0.72	<0.0001
Stage 5 CKD population						
ESRD* (event number/total n)						
Control (3/3)	1.00	Reference		1.00	Reference	
PPI cohort (7/12)	0.80	0.36-1.82	0.60		Not converged	
H2RA cohort (0/0)	-	-	-	-	-	-
Overall mortality# (event number/total n)						
Control (1/3)	1.00	Reference		1.00	Reference	
PPI cohort (5/12)	1.71	0.46-6.30	0.42		Not converged	
H2RA cohort (0/0)	-	-	-	-	-	-

Abbreviations: the same as Tables 1-2.

*Adjusted for all covariates (age per year, sex, comorbidities, number of medical visits, NSAID, and ACEI/ARB) and competing mortality.

#Adjusted for all covariates (age per year, sex, comorbidities, number of medical visits, NSAID, and ACEI/ARB).

Table S6. Association between 5, 10, 15, and 20 of cumulative define daily dose (cDDD) of H2RA and study outcomes

	ESRD			Overall mortality			CV mortality			Non-CV mortality		
Taking controls as the reference	events (%)	aHR* (95% CI)	*p*-value	events (%)	aHR# (95% CI)	*p*-value	events (%)	aHR# (95% CI)	*p*-value	events (%)	aHR# (95% CI)	*p*-value
cDDD≤5 (n=1 548)	33 (2.1)	0.48 (0.29-0.79)	0.004	246 (15.9)	1.12 (0.91-1.39)	0.27	52 (3.4)	2.03 (1.12-3.69)	0.02	194 (12.5)	1.03 (0.82-1.29)	0.82
5<cDDD≤10 (n=778)	17 (2.2)	0.39 (0.19-0.79)	0.009	89 (11.4)	0.84 (0.62-1.13)	0.25	13 (1.7)	1.18 (0.49-2.84)	0.72	76 (9.8)	0.79 (0.57-1.10)	0.16
10<cDDD≤15 (n=490)	6 (1.2)	0.43 (0.15-1.23)	0.12	59 (12)	0.53 (0.35-0.80)	0.002	6 (1.2)	0.40 (0.14-1.20)	0.10	53 (10.8)	0.56 (0.36-0.87)	0.009
15<cDDD≤20 (n=260)	6 (2.3)	0.68 (0.18-2.59)	0.58	31 (11.9)	0.54 (0.31-0.92)	0.025	3 (1.2)	0.29 (0.06-1.43)	0.13	28 (10.8)	0.59 (0.33-1.05)	0.07
20<cDDD (n=1 285)	19 (1.5)	0.24 (0.13-0.42)	<0.0001	206 (16)	0.48 (0.39-0.59)	<0.0001	20 (1.6)	0.53 (0.28-1.00)	0.05	186 (14.5)	0.47 (0.38-0.59)	<0.0001

Abbreviations: CV, cardiovascular; the same as Tables 1-4.

*Adjusted for all covariates (age per year, sex, comorbidities, number of medical visits, NSAID, and ACEI/ARB) and competing mortality.

#Adjusted for all covariates (age per year, sex, comorbidities, number of medical visits, NSAID, and ACEI/ARB).

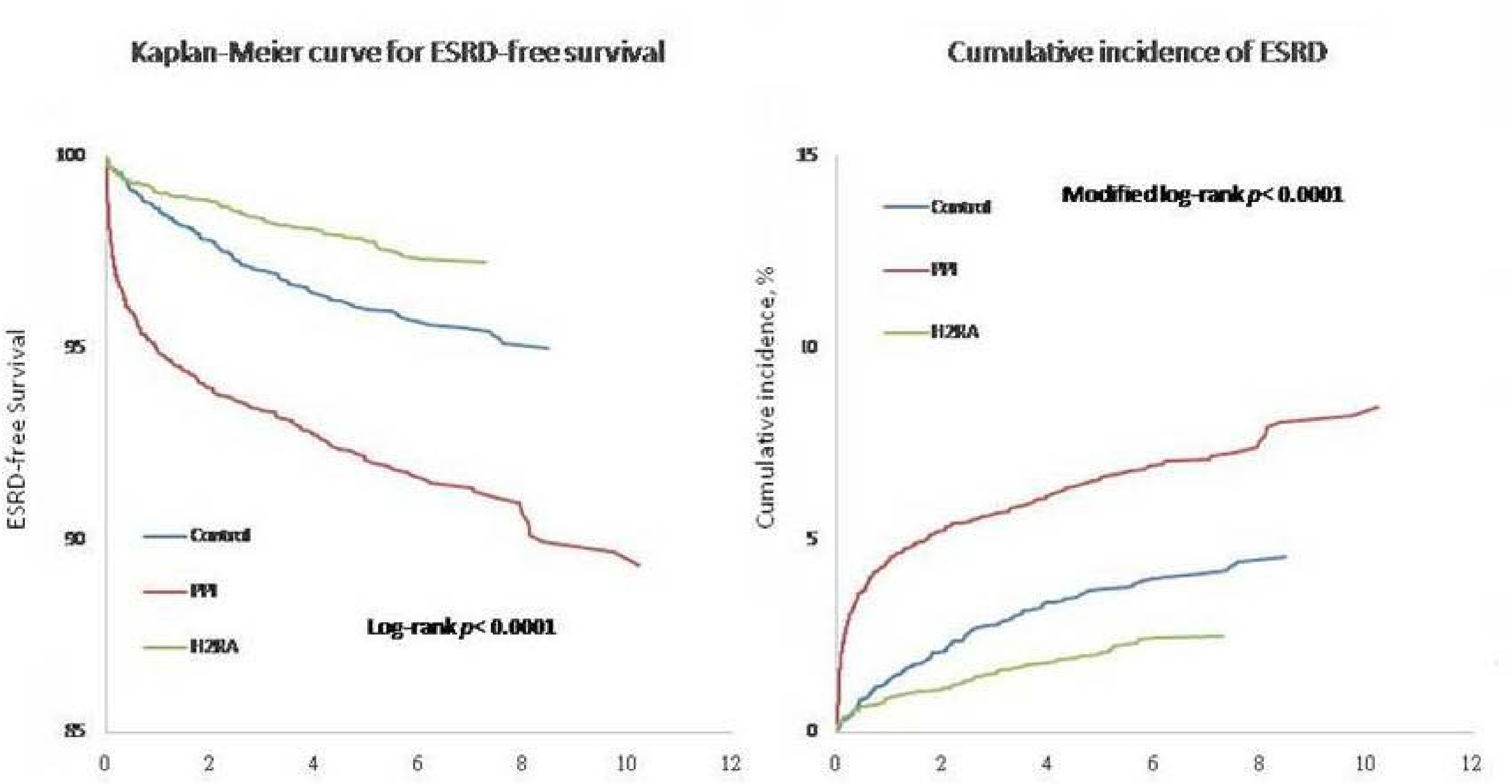

Figure S1. (Left) Kaplan-Meier curve for ESRD-free survival between H2RA, PPI, and control cohorts. (Right) The cumulative incidence of ESRD was estimated in consideration of the competing risk of mortality, and the differences between H2RA, PPI, and control cohorts were analyzed using modified Kaplan–Meier and Grey methods.

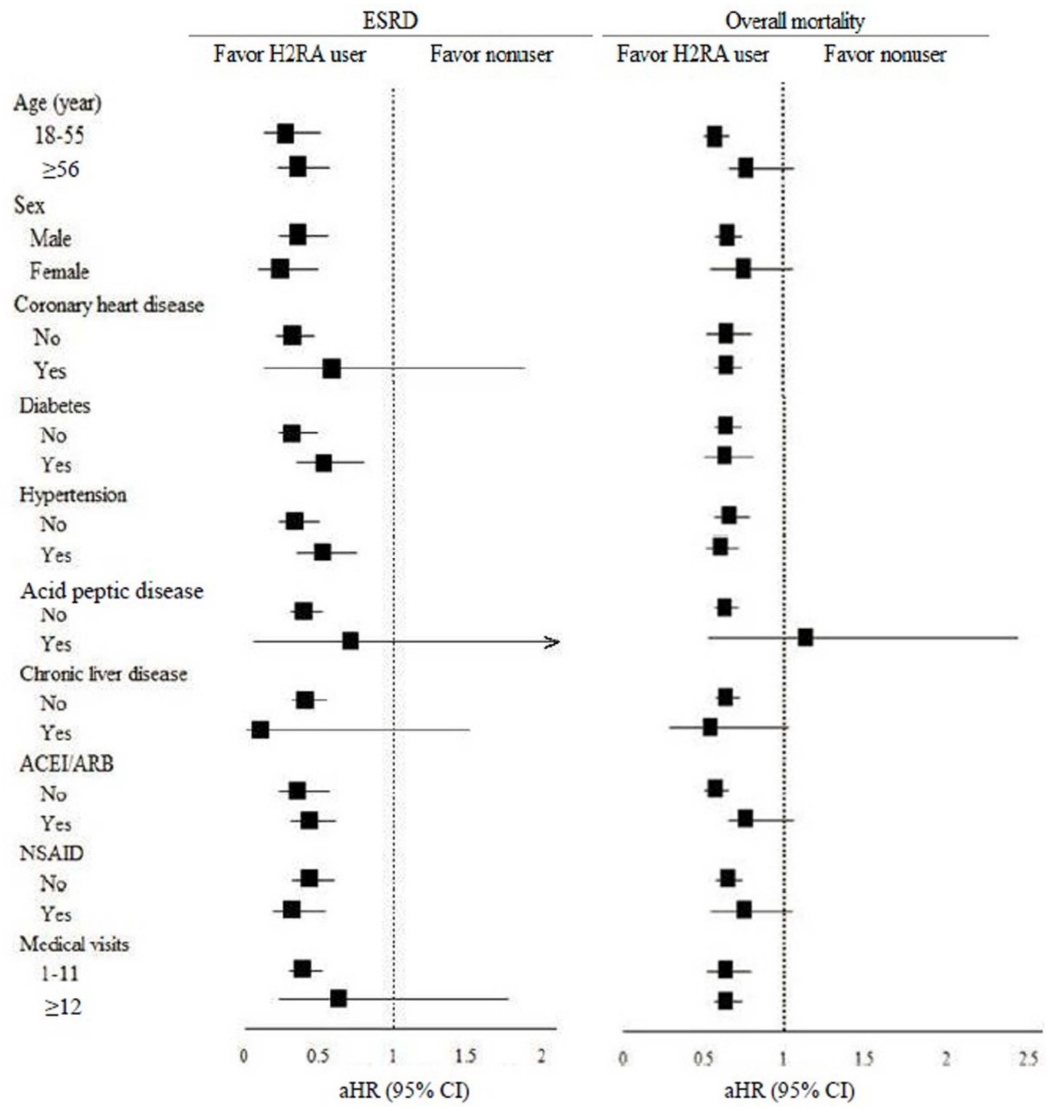

Figure S2. Multivariate stratified analyses for the association between H2RA use (*vs.* nonuse) and risks of ESRD and overall mortality. Each factor was adjusted for all other factors listed in Table 1.
